# Biosensing Using Magnetic Particle Detection Techniques

**DOI:** 10.3390/s17102300

**Published:** 2017-10-10

**Authors:** Yi-Ting Chen, Arati G. Kolhatkar, Oussama Zenasni, Shoujun Xu, T. Randall Lee

**Affiliations:** Department of Chemistry and the Texas Center for Superconductivity, University of Houston, Houston, TX 77204, USA; ychen75@uh.edu (Y.-T.C.); kolhatkara@yahoo.com (A.G.K.); Zenasni_sam@hotmail.com (O.Z.)

**Keywords:** magnetic particles, spintronic sensors, GMR, NMR, SQUID, atomic magnetometer, molecular sensing

## Abstract

Magnetic particles are widely used as signal labels in a variety of biological sensing applications, such as molecular detection and related strategies that rely on ligand-receptor binding. In this review, we explore the fundamental concepts involved in designing magnetic particles for biosensing applications and the techniques used to detect them. First, we briefly describe the magnetic properties that are important for bio-sensing applications and highlight the associated key parameters (such as the starting materials, size, functionalization methods, and bio-conjugation strategies). Subsequently, we focus on magnetic sensing applications that utilize several types of magnetic detection techniques: spintronic sensors, nuclear magnetic resonance (NMR) sensors, superconducting quantum interference devices (SQUIDs), sensors based on the atomic magnetometer (AM), and others. From the studies reported, we note that the size of the MPs is one of the most important factors in choosing a sensing technique.

## 1. Introduction

Magnetic particles (MPs) in the nanometer to micrometer size range are used extensively in modern bioanalytical methods and biomedical therapies. Magnetic detection offers the distinct advantage of low background interference compared to optical and electrical approaches [1,2]. Due to their facile functionalizability, MPs have the potential of integrated design for a wide range of purposes, such as signal markers, separation platforms, force transducers, and sensing devices. Additionally, MPs have been used as imaging agents, heat generators, and drug carriers [1,3,4]. Moreover, the ability to measure biological interactions using a magnetically labeled target (i.e., a ligand-receptor complex) furthers our understanding of the interactions between biomolecules, which allows advancements in the design of emerging diagnostic techniques.

In general, designing MPs for a specific application involves several processes, including: (i) synthesizing MPs of a suitable size, (ii) modifying the MPs with a biocompatible linker and suitable ligands, (iii) tailoring the size of the MPs and ligands to optimize the magnetic properties for the selected application, and (iv) identifying an optimal magnetic detection technique to test and use the MPs. Current studies have provided us with parameters to synthesize MPs selectively from the nanometer to the micrometer size range. For in vivo biomedical imaging and drug delivery, the size of the MPs should be below 100 nm to ensure endocytosis into target organs and cells [5]. However, for biological sensing, larger MPs give stronger signals owing to their higher magnetization [4] and are preferred. Additionally, surface functionalization is critical to protect the magnetic core and to attach it to target molecules. Numerous studies have reported the effect that coating MPs with shells and functional ligands have on generating successful biomedical applications [3,6,7,8].

The last step in the design of a MP system is selecting an analytical technique to detect the magnetic signal. There are several types of magnetic detection techniques, including but not limited to spintronic sensors based on giant magnetoresistance (GMR), tunnel magnetoresistance (TMR), and planar Hall effect (PHE) sensors, superconducting quantum interference devices (SQUIDs), atomic magnetometers (AMs), nuclear magnetic resonance (NMR) systems, fluxgate sensors, Faraday induction coil sensors, diamond magnetometers, and domain walls-based sensors [3,9,10,11,12,13,14,15,16,17,18,19]. These techniques have been used to measure the magnetic response in the form of susceptibility, relaxation, remanence, MP-induced proton NMR, and even frequency mixing [17,20,21,22,23,24,25,26]. Also, these detection techniques can be categorized as either volumetric-based or surface-based [6]. The volumetric-based sensors, such as PHE sensors and NMR systems, provide simple and rapid sample preparation and detection. Separately, surface-based sensors, such as GMR and TMR, offer a lower detection limit (single particle) due to the short distance between the MPs and the sensor; however, these techniques typically require laborious sample and/or substrate preparation. Sensitive magnetometers, such as SQUIDs and AMs, can be used in either mode, depending on the specific applications. To date, several types of magnetic sensors have been developed and integrated with various other techniques to provide biosensing for a wide range of applications [12,13,14,25]. Nevertheless, optimizing MPs for specific applications and selecting appropriate detection methods remain challenging for the magnetic nanotechnology community due to the increasing demands of detection sensitivity, molecular specificity, and application complexity. Our report describes the synthesis and functionalization of MPs and their subsequent use for detection. To provide context, we briefly discuss the basic concepts in magnetism and highlight the critical magnetic parameters that can be manipulated based on the application targeted.

## 2. Basic Concepts in Magnetism and Use of Magnetic Particles in Biological Applications

**Figure 1:**
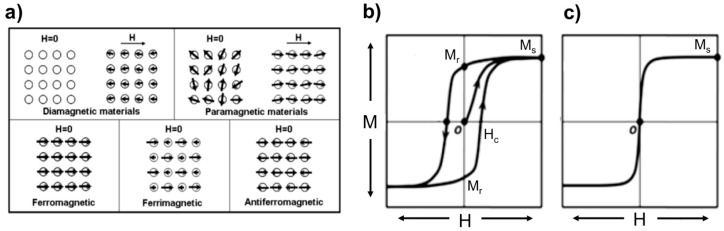
(**a**) Illustration of the behavior of the magnetic dipole moments in five types of magnetic materials with and without an external magnetic field (H). (**b**) Schematic of typical magnetization curves generated by an applied magnetic field. The general hysteresis loop shows the relationship of magnetization versus the applied magnetic field. The curved slope defines the magnetic susceptibility of the material. (**c**) A non-hysteretic magnetic curve of a superparamagnetic material. Reproduced with permission from [28], copyright 2007 Wiley, and from [8], copyright 2009 The Royal Society of Chemistry.

The magnetic properties of nano- and micron-sized magnetic materials differ from those of the corresponding bulk magnetic materials. Typically, MPs are classified (see Figure 1a) as paramagnetic, ferromagnetic, ferrimagnetic, antiferromagnetic, or superparamagnetic based on their magnetic behavior in the presence and absence of an applied magnetic field [27,28,29].

Figure 1b illustrates the fundamental magnetic properties of magnetic materials, including magnetic particles. In the presence of an external magnetic field, the magnetic moment of the material is aligned along the direction of the external field, where the maximum magnetization is labeled as the saturation magnetization (M_s_). At zero magnetic field, the magnetization of these materials tends to retain the previous direction and magnetization, called the “remanent magnetization” (M_r_). Reversing the direction of the external magnetic field brings the ferromagnetic domains into a compensated state, and the overall effective magnetization appears to be zero. The field strength required to demagnetize the MPs (forcing the magnetization to be zero) is defined as the coercivity (H_c_). Figure 1c shows a unique paramagnetic feature of MPs called superparamagnetism. For a single domain MP at a sufficiently high temperature (i.e., the blocking temperature), there is sufficient thermal energy to overcome the anisotropy barrier, which randomizes the magnetization. Therefore, in the absence of external magnetic fields, MPs exhibit negligible remanent magnetization. However, with increasing external magnetic fields, the magnetization of the MPs grows, similar to paramagnetic materials [8,29].

MPs have been used in a variety of biological applications [4,29]; herein, we review and correlate certain applications to the critical magnetic properties, such as, M_s_, H_c_, blocking temperature, and magnetocrystalline anisotropy [27]. One of the key factors in magnetic biosensing is magnetic susceptibility for small fields and saturation magnetization (M_s_) for large fields: a stronger magnetic moment (high M_s_) of labeled MPs offers higher sensitivity toward a sensor platform fabricated with its conjugate biomolecule. The M_s_ in MPs is also related to the translational vector for spatial movements, where higher M_s_ provides a stronger translational attractive force when exposed at a large magnetic gradient [4]. Moreover, a larger M_s_ often corresponds to a larger mass for the MPs. Several multimodal techniques have been developed that treat MPs as both force transducer and signal provider. For example, the force-based technique of magnetic tweezers and force-induced remanent magnetization spectroscopy (FIRMS) use MPs to induce dissociation of ligand-receptor pairs and then quantify the magnetic signal [25,30].

As noted above, the size of particles also plays an important role in determining their magnetic properties. The M_s_ of MPs usually increases with an increase of particle size due to a decrease in the surface effect in which the spins on the surface can differ from those in the bulk [4,31]. Furthermore, the homogeneity of MPs is uniquely important in determining the translational force for the separation of biomolecules and magnetic manipulation in sensing applications [32,33,34]. MPs can be held in a saturated state only by an external field larger than the demagnetizing field. Once this external field is removed, the magnetostatic energy associated with the saturated state breaks the particles into domains and reduces the magnetization [11,29,35]. For most magnetic materials, the single domain size usually falls in the range of 10–50 nm and is related with their intrinsic properties (such as magnetization saturation, magnetic anisotropy constant, and the exchange stiffness) [1]. When the size of MPs becomes smaller than one single domain, the MPs become superparamagnetic. In large MPs composed of multi-domain structures, the magnetization separated by domain walls is retained.

Relaxation is a unique magnetic phenomenon when the magnetic dipoles lose their effective moments. This phenomenon occurs via two different relaxation mechanisms: (i) Néel relaxation, which originates from spin-spin relaxation through the rotation of the magnetic moment within the MPs overcoming the anisotropy energy barrier, and (ii) Brownian relaxation, which comes from the mechanical rotation of the MPs. Relaxation measurements are commonly used in magnetic detection. Typically, Néel relaxation is more dominant for small MPs (range < 15 nm for iron oxide) and depends on the magnetocrystalline anisotropy [2,27,36].

Relaxation processes occurring in MPs is harnessed in magnetic hyperthermia therapy applications. In this therapy, the generation of heat occurs either by repeated Néel and Brownian relaxation processes or by hysteresis loss when the MPs are exposed to an alternating magnetic field. The heat is then transferred to the surroundings, which provide sufficient energy to treat, for example, targeted tumor tissue. The generation of heat is highly sensitive to the magnetic properties, such as M_s_, H_c_, magnetic anisotropy (K), particle size, and surrounding environment [1,4,36]. In drug-delivery applications, the MPs are visualized using magnetometer imaging and then guided in the presence of an external magnetic field; the heat is used to trigger drug release or for tissue ablation. The external magnetic field decays with distance, and high-M_s_ MPs are preferred since they can be easily magnetized in a weak field [4].

MPs are also used to alter the relaxation of nuclear spins, based on which NMR technique is used to detect the MPs. When exposed to an external magnetic field, MPs create small local magnetic fields that make the surrounding protons undergo faster spin-spin relaxation times (T_2_). The critical magnetic parameter for imaging is the transverse relaxation rate (R_2_), which is defined as R_2_ = 1/T_2_. It has been demonstrated that MPs can induce high T_2_ relaxivity, which is defined as the R_2_ difference in the presence vs. the absence of the MPs divided by the particle concentration [4,37]. Higher relaxivities can be achieved using MPs with larger sizes and higher M_s_ values [38]. MPs as contrast agents in magnetic resonance imaging (MRI) studies and as labeling and signal transducers in biosensing studies are excellent examples of the effect of MPs on nuclear spin relaxation [2,7,37,39,40].

## 3. Synthesis, Functionalization, and Biological Conjugation of Magnetic Particles

**Figure 2:**
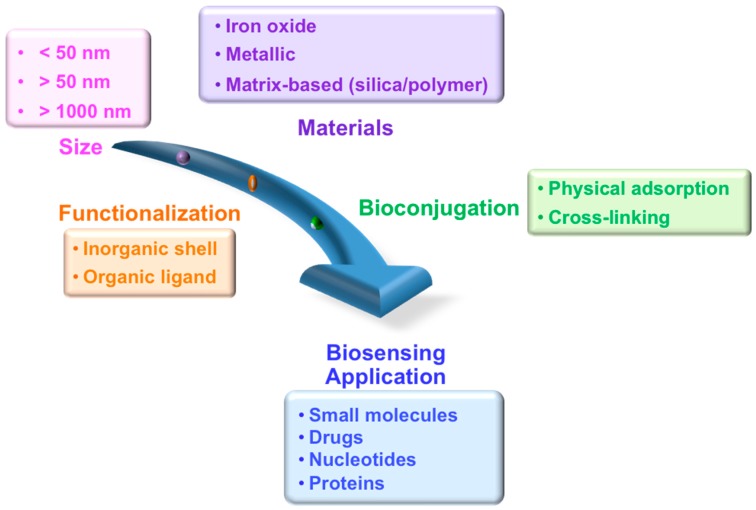
Design of magnetic particles for biosensing.

After highlighting the importance of various magnetic properties and the role of size as well as composition, this section summarizes factors that are evaluated during the design of appropriate MPs for specific bio-applications (Figure 2). The process involves (1) the synthesis of MPs having a desired size, (2) functionalization with biocompatible ligands having functional or reactive groups, and (3) conjugation with the target biological species. The key parameters include the size of the MPs (both core and hydrodynamic sizes), chemistry of surface functionalization, and the magnetic response to the sensor. In this section, we describe the synthesis, functionalization, and bioconjugation steps in detail.

### 3.1. Synthesis of Magnetic Particles

The synthesis of various types of MPs has been extensively studied [2,7,8,41]. We will focus on the two main types of MPs: (1) single-core MPs (ferrites and other metallic cores), and (2) matrix-dispersed MPs. Table 1 summarizes the different methods used to synthesize MPs as a function of their sizes and compositions.

#### 3.1.1. Single-Core Magnetic Particles

**Ferrites:** Iron oxide MPs, such as magnetite (Fe_3_O_4_) and maghemite (γ-Fe_2_O_3_), are particularly appealing due to their magnetic properties, tunable size, and biocompatibility [37,41,74,75]. Numerous chemical methods have reported the synthesis of iron oxide and metal-substituted ferrite MPs. Commonly used wet chemistry methods of synthesis include co-precipitation, thermal decomposition, and hydrothermal.

Co-precipitation processes utilize selected stoichiometric amounts of ferrous (Fe^2+^) and ferric (Fe^3+^) salts in an alkaline solution in conjunction with a water-soluble surface coating material, such as polyethylene glycol (PEG), where the coating provides colloidal stability and biocompatibility. The size and properties of the MPs can be controlled by tuning the reducing agent concentration, pH, ionic strength, temperature, iron salts source, or Fe^2+^/Fe^3+^ ratio. However, this approach leads to relatively large size distributions [3].

In the thermal decomposition method, organometallic complexes and metal precursors such as iron cupferronate, iron acetylacetonate, and iron oleate are decomposed at elevated temperatures [48,49,50]. The size and shape of MPs can be tailored by varying the reaction conditions, such as the type of organic solvent, heating rate, surfactant, and reaction time. This method leads to narrow size distributions of MPs in the size range 10–100 nm [41,49] and is especially useful since it can be extended to the synthesis of metal-doped ferrite MPs (MFe_2_O_4_, M = Co, Fe, Ni, and Zn) by using additional divalent transition metal precursors. The substitution of Fe^2+^ by other metals offers an opportunity to boost the saturation magnetization [51]. The presence of a hydrophobic coating during the synthesis process warrants an additional step of ligand exchange so the MPs can be dispersed in water for further uses.

The hydrothermal method involves liquid-solid-solution phase transfer for the synthesis of variety of nanoparticles including iron oxide nanoparticles [62]. As in the case of thermal decomposition, the polyol-hydrothermal reduction method leads to water-dispersed MPs in the size range from tens to several hundred nanometers [64]. The solvent system, reducing agent, and type of surfactant are some of the parameters that can be used to tune the size and the surface-functionalization of iron oxide MPs [65]. In the reverse water-in-oil micelle method, which involves aqueous nanodroplets of iron precursors, a microemulsion is formed that is stabilized by a surfactant in the oil phase. Then magnetic nanoparticles can be obtained by precipitation [68,69]. The combination of microemulsion and silica sol-gel has been reported to assemble small iron oxide nanocrystals, which are obtained via co-precipitation into larger magnetic particles of more than 100 nm in diameter [76].

**Metallic (Elemental):** Metallic MPs possess high saturation magnetization and are either monometallic (e.g., Fe, Co, or Ni) or bimetallic (e.g., FePt and FeCo). These metallic MPs are often overlooked in biological applications due to their chemical instability and their toxicity; they typically require an external coating to overcome these disadvantages. The thermal decomposition of metal precursors and metal reduction routes are widely used for the synthesis of metallic MPs and bimetallic MPs [53,54]. For example, a common route employed for the synthesis of iron oxide MPs involves the thermal decomposition of iron pentacarbonyl (Fe(CO)_5_) [55]. The synthesis of cobalt MPs utilizes the reduction of dicobalt octacarbonyl (Co_2_(CO)_8_) by Al(C_8_H_17_)_3_ in toluene [53].

Popular alloy MPs, including FeCo and FePt, have found use in biological applications [2,39,56]. Physical methods for the synthesis of alloy MPs include vacuum-deposition and gas-phase evaporation. However, physical methods suffer from certain limitations, such as aggregation, wide size distribution, and poor colloidal stability [77]. In contrast, wet chemical synthesis offers an effective route for the preparation of monodisperse bimetallic MPs. For example, FeCo MPs with high saturation magnetization (reported 207 emu/g) are suitable for biomedical applications and can be synthesized via the reduction of Fe^3+^ and Co^2+^ salts [53,57]. Furthermore, FeCo nanocubes can be obtained by the liquid-phase reduction of Fe^2+^ and Co^2+^ by hydrazine in the presence of poly(ethylene glycol) and cyclohexane to give sizes tunable from 68 to 260 nm edge length [78,79]. For FePt MPs, the most common method involves the thermal decomposition of iron pentacarbonyl (Fe(CO)_5_) or thermal reduction of iron salts (e.g., iron(II) chloride) combined with the reduction of platinum(II) acetylacetonate (Pt(acac)_2_) to give MPs with diameters that range from 3 to 9 nm [56,58]. By varying the ratio of stabilizer (oleylamine, oleic acid, or octadecene) to metal precursor, the shape of the FePt MPs can be tuned to nanowires, nanorods, or nanocubes [59,60]. Another synthesis route to obtain FePt MPs proceeds via the polyol process, which involves the co-reduction of the metal precursors Fe(acac)_3_ and Pt(acac)_3_ in 1,2-hexadecanediol [80].

#### 3.1.2. Matrix-Dispersed Magnetic Particles

**Figure 3:**
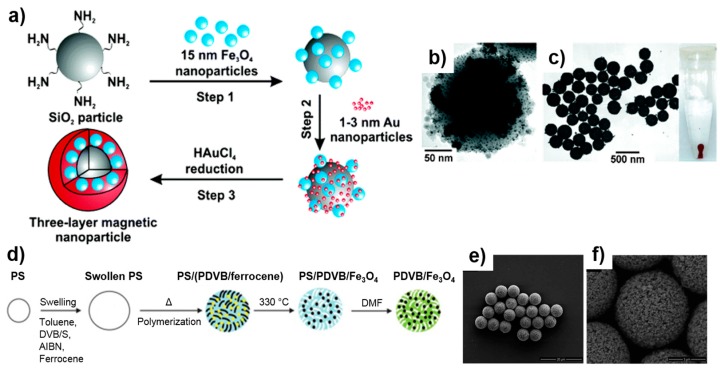
Examples of preparation methods for MPs embedded in silica and polymer matrices: (**a**) layer-by-layer synthesis of gold-coated silica-core MPs, (**b**) TEM image of SiO_2_@Fe_3_O_4_@Au_seeds_, (**c**) MPs attracted to the vessel wall by a magnet, (**d**) preparation of polymer composite magnetic particles by the swelling method, and (**e**,**f**) SEM image of polymer-embedded MPs. Reproduced with permissions from [72], copyright 2005 American Chemical Society, and from [73], copyright 2012 Royal Society of Chemistry.

In addition to single metallic or metallic oxide core MPs, multi-core MPs, multilayers of magnetic materials and nonmagnetic materials, as well as the coating of silica or polymer cores with magnetic shells have also been reported [81]. Commonly used nonmagnetic core particles include silica and various polymers. These cores, in comparison to metallic oxide or metallic MPs, provide several advantages including narrow size distributions within a wide range of possible sizes, high chemical stability, and facile functionalization with various targets. One type of architecture consists of a dielectric silica core coated with a magnetic shell (Co, FePt, or Fe_3_O_4_) together with a stabilizer (silica shell or polyelectrolyte layer) [70,71,72]. For example, the layer-by-layer synthesis of gold-coated silica-core magnetic MPs is shown in Figure 3a–c [72]. Mesoporous MPs, consisting of a silica/polymer framework with encapsulated MPs, is another useful design for multifunctional applications combining MR imaging, optical fluorescence imaging, cell targeting, and drug-delivery capability [82]. The microemulsion method was used to synthesize such silica particles loaded with iron oxide MPs via reverse micelles, and polymer particles containing iron oxide aggregates via swelling processes [66,73]. Magnetic materials embedded into a polymer matrix using the latter approach offers good size control up to 6 μm in diameter. Figure 3d–f gives an example of the swelling procedure used for the synthesis of poly(divinyl benzene) infused with MPs [73]. Commercially available, matrix-dispersed MPs include styrene-based particles with 5–20% of crystalline iron oxide [83]. The performance and sensitivity of these commercial magnetic microspheres in various applications has been demonstrated using magnetic detection techniques [12,25,84].

#### 3.1.3. Toxicity

Due to the potential applications of MPs in biomedical and biological research, the toxicity of MPs is a concern that must be addressed. Since most of the MPs are composed of transition metals, the leaching and biodegradation of MPs are likely to cause the release of free metal ions, leading to the generation of reactive oxygen species (ROS) and their related undesirable effects. Exposure to MPs can lead to (in addition to the generation of ROS) inflammation, impaired mitochondrial function, DNA damage, and severe toxicity at ~60 mg iron/kg animal [85,86]. Notably, ROS can trigger undesired defense mechanisms in the body, which are commonly considered to be the main cause of toxicity from MPs. The coating on MPs can inhibit the ROS process [87,88]; for example, iron oxide MPs can be rendered biocompatible with suitable coating materials, such as silica, gold, or PEG.

The toxicity of MPs depends on the size, chemical composition, surface charge, and particle-induced reactions. Smaller MPs typically have a larger surface area-to-volume ratio and are chemically more reactive than their larger counterparts [1,89]. Hydrodynamic size is another parameter that affects the functionality and permeation in biological media. The surface charge and coating can alter the hydrodynamic size, which can be tuned to optimize functionality. In addition, the interface between MPs and biological systems is one of the critical factors influencing the biosafety of nanoparticles [82,89]. In short, designing a suitable surface coating on the MPs of chosen size and shape will define the interactions between the particles and biological molecules and their biocompatibility.

### 3.2. Functionalization of Magnetic Particles

The stabilization of MPs is crucial for any application. Preventing agglomeration or precipitation stands as a challenge due to the attractive van der Waals and magnetic inter-particle forces. Charged and/or bulky ligands can be used to provide forces of electrostatic and steric repulsion, respectively, for the MPs. Such coatings not only protect the magnetic cores, but also can render them biocompatible (minimizing toxicity effects), while also providing a chemical handle for the further conjugation of affinity ligands, such as proteins or DNA. Various types of inorganic and organic functionalization strategies are described below.

#### 3.2.1. Inorganic Shell Coatings: Silica and Gold

Inorganic materials as protective shell coatings have become an attractive approach to shield against environmental influence. The most common approach to encapsulate the magnetic nanoparticles is by using a core-shell structure comprised of biocompatible silica or gold to cover the magnetic nanoparticles [39]. These popular coating materials provide protection against chemical oxidation or degradation of the magnetic core and prevent potentially toxic leakage of metal ions from the magnetic core. Additionally, the shell provides a useful platform to modify the particles with various ligands (e.g., the gold shell binds to thiols and related compounds). Other inorganic materials used as coatings include titanate or silver [90,91,92]. For example, silver-coated iron oxide MPs were synthesized and integrated with carbon paste for use as an immunosensor [92]. In this review, we have focused on silica and gold shells due to the ease and versatility in their ensuing functionalization.

**Silica:** Silica shells have been widely exploited due to the convenience of the coating procedure and subsequent functionalization. The synthesis of silica-coated magnetic nanoparticles by the Stöber method using sol-gel principles has been known for many years [93]. Typically, tetraethoxysilane (TEOS) is used to produce the coating on various magnetic cores with controllable thicknesses [94]. To deposit silica on MPs, TEOS hydrolysis under basic conditions (e.g., aqueous ammonium hydroxide solution) is performed to condense and polymerize TEOS into a silica shell on the surface of the magnetic core. The coating thickness can be controlled by adjusting the relative concentrations of ammonium hydroxide, TEOS, and water, or by using ultrasound treatment [95,96]. The thickness of the silica coating influences the relaxation properties of the MPs [42]. Polyvinylpyrrolidone (PVP) is a common agent used in the coating procedure to stabilize the MPs in aqueous solution and to prevent agglomeration and multicore coating [42,96,97]. Alloy magnetic nanoparticles, FeCo and CoPt, have also been successfully coated with silica [61,79]. A modified Stöber method combining (3-aminopropyl)trimethoxysilane (APTMS) and TEOS was used to coat silica on cobalt MPs [98].

Another popular approach for coating with silica is the Philipse method, in which a silica shell is formed by depositing sodium silicate on the magnetic core [43]. However, since the first layer of silica coating is usually too thin, a second layer of silica is deposited via utilization of the Stöber method [43,44,99]. Yang and co-workers, for example, prepared silica-coated, multi-core iron oxide MPs via the Philipse and Stöber methods under ultrasonic treatment [44]. Separately, the reverse microemulsion method is another popular strategy for coating with silica and has been used with surfactants such as Igepal CO-520 to give silica shell thicknesses in the range of 5–20 nm [100].

One of the greatest advantages of coating with silica is the ability of the silica-coated MPs to bind covalently with versatile functional molecules and surface-reactive groups. Important reagents include amino-terminated silanes, such as (3-aminopropyl)trimethoxysilane (APTMS), and alkene-terminated silanes, such as (3-methacryloxypropyl)trimethoxylsilane, which are then available to grow a polymeric shell on the surface of silica-coated MPs through crosslinking polymerization [33,99]. However, a disadvantage of the silica shells and/or silane ligands is their instability under harsh conditions, where Si-O-Si bonds are readily hydrolyzed under basic conditions [82,101,102]. Importantly, this drawback can turn to an advantage by developing a mesoporous silica shell through careful control of the basic conditions and by adding a surfactant, such as cetyl trimethylammonium bromide (CTAB). The mesoporous silica coating can be used as a drug-delivery or enzyme-delivery cavity; it offers the ability to load and release species such as drugs or proteins in MPs with tunable silica pore sizes [101,102,103,104]. For example, Yue and co-workers synthesized mesoporous magnetic microspheres and demonstrated the immobilization of different sizes of enzymes [101].

**Gold:** Gold as a protective shell is inert and biocompatible, and it has a unique binding specificity with alkanethiols [105,106]. Gold shells provide additional functionality in diagnostics and therapeutics via the strong absorption of light in the visible and near infrared [106,107,108]. The surface plasmon effect offers multifunctional therapeutic heating for gold-coated MPs [103,106,107,108]. Notably, the synthesis of gold-coated iron oxide nanoparticles can be accomplished using chemical methods, reversed microemulsion, and laser-promoted methods [81,107]. Gold-coated MPs are prepared either by directly coating gold on the MP core or by using silica as an intermediate layer for gold coating [81,108].

Reduction is the most commonly used method to deposit gold shells on MPs. Metal oxide magnetic cores or silica-coated magnetic cores are first functionalized with APTMS, and then 2–3 nm gold nanocrystal seeds from chloroauric acid (HAuCl_4_) are electrostatically attached to the surface. Then mild reducing agents, such as sodium citrate or tetrakis(hydroxymethyl)phosphonium chloride (THPC), effect the reduction of HAuCl_4_ on the MP oxide surface, giving rise to the gold shell [90,107,108]. The compound, gold(III) acetate (Au(OOCCH_3_)_3_), serves as an alternate gold source for this reduction method [105]. Several bio-applications, such as MRI, fluorescence imaging, and catalysis are based on gold-coated MPs [106,109]. Reverse micelles have also been used to grow gold shells on metallic magnetic cores, such as nickel and iron [67,110].

**Figure 4:**
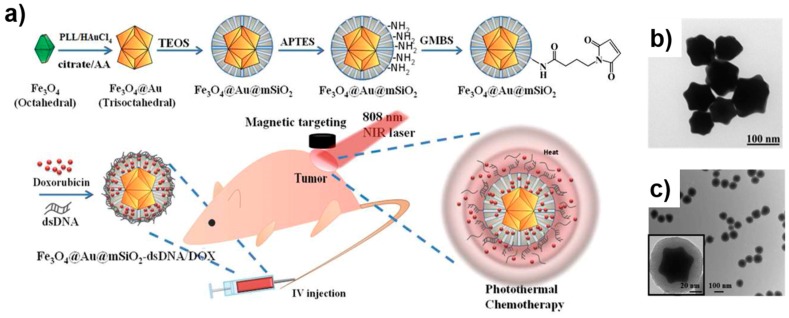
(**a**) Illustration of the preparation of Fe_3_O_4_@Au@mSiO_2_-dsDNA/DOX nanoparticles for in vivo treatment of cancer cells, (**b**) TEM image of Fe_3_O_4_@Au, and (**c**) TEM image of Fe_3_O_4_@Au@mSiO_2_. Reproduced with permission from [103], copyright 2014 American Chemical Society.

The multiple advantages of silica and gold shells have attracted researchers to develop multifunctional MPs [103,104]. Figure 4 illustrates the various projected applications of multifunctional Fe_3_O_4_@Au@mSiO_2_ MPs [103].

#### 3.2.2. Functionalization with Organic Ligands

The functionalization of MPs with organic ligands can be performed in-situ or post-synthesis. The in-situ method provides functional ligands on MPs during the synthesis step, and the MPs are immediately ready for further conjugation and use [111]. For example, MPs can be tethered with terminal hydroxyl groups (-OH), amino groups (-NH_2_), and carboxyl groups (-COOH) by varying the surfactant (e.g., dextran, chitosan, or poly(acrylic acid)) used in the hydrothermal synthesis [111]. However, the in-situ method cannot be applied universally for all possible ligands. In contrast, the post-synthesis method allows functionalization of customized ligands on almost any MP surface.

Ligand addition and ligand exchange are the two primary strategies for the post-synthesis functionalization of MPs. Ligand addition can be achieved through the adsorption of amphiphilic molecules that contain both a hydrophobic segment and a hydrophilic component to form a double-layer structure [8]. On the MP surface, the metallic atoms are electron deficient; consequently, the surfaces have an affinity for electron-rich functional groups such as amines, carboxylates, hydroxyls, phosphates, and thiols. The ligand-exchange process directly replaces the original surfactants (or ligands) with new functional ligands. The new ligands usually contain a functional group that is capable of binding on the MP surface via either strong chemical bonding or electrostatic attraction, and another functional group for stabilization in water and further bio-functionalization. For hydrophobic MPs, ligand exchange is the main approach to enhance MP dispersibility in water by replacing the hydrophobic capping ligands with hydrophilic ligands, such as polyethylene glycol (PEG) or polyvinylpyrrolidone (PVP) or selected organosilanes and thiols [42,46,52]. For example, the hydrophobic ligands on 6 nm Fe_3_O_4_ MPs were replaced with organosilane terminated with -NH_2_, -SH, and -COOH groups for further conjugation with fluorescent dyes [46]. Another study utilized nine types of silane ligands (terminated with amine, short aldehyde, acrylate, isocyanate, thiol, PEG, long aldehyde, cyano, or carboxylate groups) and exchanged with oleic acid present on the CoFe_2_O_4_ MPs [52]. We have broadly classified the ligands as “small monomeric” and “polymeric” for ease of discussion.

**Small Monomeric Ligands:** Suitable molecular species that enhance ionic stability and further chemical modification are typically chosen as ligands to adorn the MP surfaces. The advantages of small molecules as ligands include tunable coverage on the MPs, a small hydrodynamic radius that is necessary for endocytosis, and a simple reaction procedure. However, small molecule ligands must be carefully evaluated since they might offer an insufficient steric barrier for colloidal stability if the molecular film is too thin. Functional groups including carboxylates, phosphates, and catechol are known to bind to the surface of metal oxides [47,112].

The siloxane group has a strong affinity for surfaces enriched in hydroxyl groups such as metal oxide MPs or silica-coated MPs. Commercially available, small silane ligands work as a bridge to link MPs and various functional ligands, including amines, carboxylates, thiols, and epoxides [33,34,102,113,114,115]; these functional ligands serve as effective linkers for further biological applications, such as protein immobilization. The silane APTMS is perhaps the ligand most widely used to modify Fe_3_O_4_ MPs, which can subsequently be conjugated with molecules such as fluorescence dyes for use in immunoglobulin (IgG) immunoassays [33]. An example of thiol-based functionalization is provided by 3-mercaptopropyltrimethoxysilane (MPTMS)-modified MPs conjugated with maleimide-functionalized streptavidin; these MPs have been used as MRI T_2_ imaging contrast agents and were used to kill HER2+ cells selectively [116]. Li and co-workers used various types of silane-functionalized MPs to conjugate with protease, followed by an evaluation of the enzymatic activity [114]. For the preparation of carboxylate-terminated MPs, the silane ligands N-(trimethoxysilylpropyl)ethylenediaminetriacetic acid (TMS-EDTA) [117] and (triethoxysilylpro-pyl)succinic anhydride have been used to functionalize the surfaces of the MPs [113,118].

The functional groups phosphonic acid and catechol provide good stability when bound to MP surfaces, and the hydrophilic tail groups offer dispersion in biological media and bioconjugation [47,112,119,120,121,122,123]. Amino-terminated phosphonic acids on iron oxide MPs have been evaluated for cellular uptake and cytotoxicity [124]; separately, multifunctional mesoporous silica MPs were functionalized with 3-(trihydroxysilyl)propyl methylphosphonate for dispersion in aqueous solution, drug delivery, MR imaging, fluorescence imaging, magnetic manipulation, and cell targeting [82]. Of the catechol-based ligands, dopamine is one of the most attractive due to the stability of its adducts [112,119]. Iron oxide MPs modified with zwitterionic dopamine sulfonate groups have demonstrated enhanced pH stability and the prevention of nonspecific interactions with proteins [32]. Several other anchoring ligands, such as dihydroxyhydrocinnamic acid, citric acid, and thiomalic acid have been used to enhance dispersion of the functionalized MPs in water [63,125,126].

**Polymeric Ligands:** Polymeric functionalization is a popular method for modifying nanoparticles because the polymer coating creates a steric barrier to prevent agglomeration and also offers the surface functionality. One concern for polymeric functionalization is the increase of hydrodynamic radius, which might hinder certain biological applications. The length and molecular weight of the polymers need to be carefully evaluated based on the intended use. The attachment of polymeric chains on MPs can occur via physical adsorption and/or covalent bonding. Both natural and synthetic polymeric ligands have been evaluated for the functionalization of MPs [127]. Natural polymeric ligands are attractive owing to their high solubility in water and low toxicity. Common natural polymers include polymeric carbohydrate-based structures such as starch, dextran, and chitosan [75,127]. Notably, dextran-coated MPs showed improved intracellular labeling when used in μNMR biosensing [128].

MPs functionalized with poly(acrylic acid) (PAA) and polyethylenimine (PEI) significantly enhanced the dispersion of MPs in water [129]. Iron oxide MPs were modified with the biocompatible polymer, poly(L-lysine), which provided a positively charged MP surface for attracting a negatively charged fluorescent agent, thus serving as a transfection agent for cancer stem cell labeling [130,131]. However, physical adsorption of the polymer is less robust than covalently attaching the polymeric ligands. Both chemically bound polymeric ligands and polymer shells are also well developed [39,75,127].

Poly(ethylene glycol) (PEG) is widely used in biomedical applications; as a nanoparticle-modifier, it offers enhanced aqueous stability, reduction in non-specific biomolecule adsorption, and increased biocompatibility due to low toxicity [118,132,133]. PEG ligands can be designed as monomeric ligands, co-polymers with versatile functionality, or as a polymeric shell [115,133,134,135,136]. The one-step of synthesis of PEG-coated MPs was reported by Lutz and co-workers, which involved the copolymer poly(oligo(ethylene glycol) methacrylate-co-methacrylic acid) (P(OEGMA-co-MAA)) as a stabilizer to obtain 10–25 nm PEGylated iron oxide MPs [133]. Post-synthesis polymer modification of MPs by the direct addition of polymer chains on MPs has been reported [134,135]. Monomeric PEG silane ligands can be used to functionalize MPs for long-term stability [118]. The self-assembly of poly(ethylene glycol)-gallol (PEG-gallol) and biotin-PEG(3400)-gallol mixed with methoxy-PEG(550)-gallol on 9 nm iron oxide MPs stabilized in aqueous solution has been demonstrated [136]. Poly(oligoethylene glycol) methyl ether acrylate and poly(dimethyl-aminoethyl acrylate) were coated on 8 nm iron oxide for protein anti-fouling and siRNA delivery [121].

In addition to PEG and natural polymeric ligands, many biocompatible and functional polymers have been customized for use as ligands on MPs. Emulsion polymerization is a common method for attaching polymers to MPs [99,102,137]. Hollow mesoporous silica MPs were prepared by growing poly(*tert*-butyl acrylate) (PTBA) chains on MPs via seed emulsion polymerization; these MPs were used as MRI contrast agents with drug-loading capacity [102]. In another example, poly(methacrylic acid) (PMAA)-coated 8 nm maghemite MPs were prepared through emulsion polymerization using potassium persulfate (K_2_S_2_O_8_) as the initiator [137]. Poly(methyl methacrylate) (PMMA)-coated Fe_3_O_4_ MPs also can be prepared through radical polymerization via the reaction between 3-methacryloxypropyltrimethoxysilane (MPS)-functionalized MPs, methyl methacrylate (MMA), and sodium lauryl benzenesulfate as the initiator [138]. The polysilane, poly(ethylene oxide)-*block*-poly(γ-methacryloxypropyl-trimethoxysilane) (PEO-*b*-PγMPS) can be directly added to MPs by silanization [139]. The self-polymerized polydopamine was functionalized on iron oxide MPs, which were used as theranostic agents for mRNA detection and image-guided photothermal therapy [140].

MPs with thick polymeric shells have been commonly prepared through distillation precipitation polymerization, which includes the initiation of polymerization from the monomer with oligomer radicals, and those oligomers crosslink to grow and precipitate from solution to form a shell [141]. Many different types of polymeric shells have been prepared with this approach, including polyacrylic acid (PAA), poly(methacrylic acid) (PMAA), poly(*N*,*N*-methylene-bisacrylamide) (PMBBAm), and poly(*N*,*N*′-methylenebisacrylamide-*co*-glycidyl methacrylate) (PMG). These polymeric shells were designed for various purposes such as biomolecular adsorption, drug loading, and delivery control [142,143,144,145]. For example, PAA-coated MPs were designed to be pH-dependent to effect controlled drug delivery [142] and to conjugate with benzoboroxole ligands for high glycoprotein capacity and low non-specific adsorption [144]. Fe_3_O_4_ MPs coated with PMG have proven useful in the highly specific separation of histidine-rich proteins [145].

### 3.3. Biomolecular Conjugation

The final step in the design of MPs for biomedical/biological applications is the conjugation of the functionalized MPs with biomolecules. The targeted biomolecules include small biomolecules, such as vitamins, peptides, and aptamers, as well as larger biomolecules such as DNA and proteins [146]. The functional group on the MP surface serves as a linker to bind with complementary biomolecules. For nucleic acid attachment, non-chemical methods (e.g., electrostatic interaction) and chemical methods (e.g., covalent bonding) are two primary means of bioconjugation. It is common to modify the nucleic acid chains with functional groups, such as thiols or amines, for facile attachment on MPs or sensor surfaces [45]. For larger biomolecules, such as proteins, their specific binding interaction with a wide range of subtracts and synthetic analogues are often employed. Proteins with specific receptor-substrate recognition, such as antigen-antibody and biotin-avidin interactions, are reliable tools in sensing applications [114,116,136,147]. A specific pair of proteins can either be used to immobilize species on MPs or on sensor surfaces to constitute the desired functionality for immunoassays or targeting studies.

Physical interactions include electrostatic, hydrophilic-hydrophobic, and affinity interactions. The primary advantage of the physical interaction method is its high efficiency without complex modification steps [7]. Several groups have demonstrated that the surface charges arising from modification with polymers (e.g., polyethylenimine, PEI) or custom ligands can be used to interact with oppositely charged biomolecules, such as positively charged MPs binding with negatively charged DNA [148,149].

The most popular affinity-based attachment method is undoubtedly the strong and specific biotin-avidin interaction. The binding of biotinylated molecules with avidin moieties is highly effective in a variety of bioconjugation systems. A wide variety of biotinylated and avidin-functionalized ligands and biomolecules are commercially available or can be prepared using unspectacular methods. Moreover, biotin molecules and tetrameric streptavidin have site-specific attraction with low nonspecific binding for controlling the direction of interacted biomolecules, such as the exposure of the Fab region of an antibody toward its antigen [146]. 

Strategies of covalent conjugation have been studied and developed over many years. Table 2 lists six strategies commonly used for covalent bioconjugation. Weissleder et al. functionalized fluorescent MPs with amino groups and linked to them different types of small molecules, such as anhydrides, carboxylates, and thiol-terminated molecules. This functionalized nanoparticle library demonstrated the ability to modulate nanomaterial surfaces for the purpose of differentiating cell lines [150]. Li and co-workers grafted MPs with six different ligands, including amines, aldehydes, carboxylates, epoxys, mercaptos, and maleimides to immobilize proteases and evaluate their stability, enzyme activity, and reusability [114]. 

Among the various versatile routes, bio-conjugation performed through the use of homobifunctional/heterobifunctional cross-linkers, carbodiimide coupling, maleimide coupling, and click reactions are well-established and offer high stability in biological systems. In the case of homobifunctional cross-linkers, glutaraldehyde and discuccinimidyl suberate (DSS) have been employed as a bridge to crosslink with amine-functionalized MPs and amine-containing biomolecules [48,147].

Carbodiimide coupling is based on the formation of an amide bond directly between a carboxylate-terminated group and an amino-functionalized molecule. In general, the carboxylate-terminated ligand is attached to the MP surface, and the free amine originates from a biological species, such as a protein. The advantages of carbodiimide coupling are the mild reaction conditions (in water at room temperature) and the fact that it leads to formation of a covalent amide bond without markedly increasing the hydrodynamic radius [127]. The most common coupling reagent is 1-ethyl-3-(dimethylaminopropyl)carbodiimide hydrochloride (EDC). The addition of *N*-hydroxysuccinimide (NHS) or sulfo-NHS increases the efficiency via formation of the transient succinimide ester. Reports of the conjugation of MPs with proteins or fluorescent dyes via carbodiimide coupling are ubiquitous in the literature [46,144].

Maleimide coupling offers a linking agent to conjugate primary amines to thiols. One approach uses amino-modified MPs to conjugate thiol-containing biomolecules, such as cysteine or herceptin. The most commonly used maleimide coupling reagent is sulfosuccinimidyl-4-(maleimidomethyl)cyclohexane-1-carboxylate (Sulfo-SMCC), which is used to activate the amino group on the MPs [114]. Another approach is the use of maleimide-modified biomolecules to crosslink with thiol-exposed ligands on the MPs. For example, the thiol group of 10 nm iron oxide MPs functionalized with (γ-methacryloxypropyltrimethoxysilane, MPTES) was conjugated with maleimide-modified streptavidin for secondary binding with biotinylated anti-HER2 [114,116].

The click reaction is a bio-orthogonal reaction that is highly selective for certain functional groups and unreactive toward functional groups commonly found in biological systems [3,120,134]. A widely used route is the Cu(I)-catalyzed alkyne-azide cycloaddition reaction, also known as click reaction CuAAC. This reaction involves the coupling of an alkyne to an azide giving a 1,2,2-triazole ring, which forms a strong covalent bond between the particle surface and the biofunctional moiety. The rapid and high-yield click reaction proceeds with high specificity between the azide and alkyne functional groups, and the resulting covalent bond is stable to harsh biological media [127]. Other types of click reactions include the Diels-Alder reaction, which in one particular case [123] involves an orthogonal clickable phosphonic acid ligand designed as two clickable parts: [3 + 2] CuAAC reaction and [4 + 2] Diels-Alder reaction. The multifunctional ligand in this example is biocompatible, has good water dispersability, and conjugates with the drug of choice attached to the thermoreversible dienophile ligand [123].

## 4. Detection Techniques and Their Applications

Once the MPs are conjugated to biomolecules as a biomarker, the final step involves the detection of the MPs. In recent years, sensing in biological and biomedical applications has been gaining significant attention and development. A broad range of magnetic detection techniques has been employed for measuring the magnetic response of the MPs. For each technique, there might be more than one magnetic parameter that can be measured. For example, SQUID magnetometers are capable of measuring relaxation, remanence, and susceptibility. To avoid substantial overlap, we categorize this section based on the different physical principles of the detection techniques instead of using both the technical basis and the magnetic parameters. Within each category, the multiple detection modes will be discussed if available.

### 4.1. Spintronic Sensors: Giant Magnetoresistance (GMR), Tunneling Magnetoresistance (TMR), Planar Hall Effect (PHE) 

Spintronic devices and their biosensing applications have been explored for decades. From this research, three types of magnetic detection techniques have emerged: giant magnetoresistance (GMR) sensors, tunneling magnetoresistance (TMR) sensors, and planar Hall effect (PHE) sensors [12,15,16,20]. Correspondingly, a wide variety of biosensing applications have been demonstrated.

Since its discovery in the 1980s, the GMR effect has been largely used in data recording [150]. More recently, GMR has found use as a magnetoresistance-based, solid-state magnetic sensor. Among the different types of sensing methods, the spin valve provides higher sensitivity with a micron-sized design [151,152,153]. A spin-valve GMR sensor consists of an artificial magnetic structure with alternating ferromagnetic and nonmagnetic layers. The magnetoresistance effect is caused by the spin-orbital coupling between conduction electrons crossing the different layers. The variation in magnetoresistance provides quantitative analysis by this spin-dependent sensor [3,6,154,155].

GMR sensors can be used for immunoassays in a manner related to enzyme-linked immunosorbent assays (ELISA). Like the sandwich-type approach in ELISA, sample preparation includes the immobilization of a molecular target on the sensor surface and the addition of tagged magnetic probes [156,157]. When the ligand-tagged MPs interact with the receptors bound to the sensor, the external magnetic dipole field from the magnetic label causes the magnetoresistance to change. The GMR sensor then detects this small magnetic signal [84].

The magnetic labels are usually fabricated as MPs. In early studies, micron-sized MPs (1–3 μm) were commonly employed as the magnetic labels [84]. However, their bulky size caused them to diffuse slowly and increased the distance of the magnetic labels from the sensor surface, inhibiting both the assay speed and detection sensitivity. Recent developments using nanometer-sized MPs as probes overcame these limitations of slow diffusion and label-sensor remoteness, which has enabled the sensitive detection of low target concentrations [158,159,160]. The smaller magnetic labels increased the density of labels bound across the sensor surface and improved the sensitivity of the sensor. For example, Xing and co-workers used high magnetic moment, cubic 12.8 nm FeCo MPs to quantify interleukin-6 (IL-6) from unprocessed human sera [160]. On the other hand, although quantification of single particles can be well defined, the size uniformity and the natural preference toward aggregation pose a challenge [158].

Importantly, magnetoresistance sensors are promising as low-cost alternatives to biochip-based biosensors [161]. Two major uses of GMR sensors include studies of DNA-DNA interactions and protein immune-sensing in which either DNAs or antibodies are used as probes to detect DNAs or proteins immobilized on the sensor surface. Current applications focus on the detection of specific DNA sequences and the improvement in detection sensitivity compared to traditional optical immunoassays [151].

GMR sensors offer significant advantages for multiplexed detection. An early GMR biosensor was developed as a bead array counter (BARC) system [162]. The integration of micron-sized MPs (2.8 μm) with the magnetoresistance sensor was able to detect biomolecular interactions such DNA-DNA hybridization [162,163]. Lately, submicron-sized magnetic markers (350 nm and 860 nm) have been used to detect DNA at concentrations ranging from 10 ng/μL to 16 pg/μL [164]. Spin-valve type GMR sensors have become popular as biochips due to their better linearity for quantification [20,153]. Fine-tuning the architectures of sensor arrays has been reported to improve the detection limit; one approach is to adjust the dimensions of sensor array for the detection of individual MPs [152]. Another approach is to use synthetic antiferromagnetic particles, which are typically fabricated as double layers of Co_90_Fe_10_ separated by a nonmagnetic spacer layer of Ru with almost zero magnetic remanence and high magnetic moment. With this approach, Fu et al. synthesized streptavidin-functionalized 100 nm synthetic antiferromagnetic nanoparticles to detect biotin-labeled DNA at a high sensitivity of 10 pM [165]. The hybrid spin-valve GMR sensor provided massive parallel sensing with a 256-sensor pre-chip array in 0.18 μm complementary metal-oxide-semiconductor (CMOS). This biosensor design monitored real-time kinetics in a protein array and detected ovarian cancer biomarkers as low as 10 fM [155]. By varying the architecture, a 64-sensor array as the circuit architecture afforded shorter readout times with a detection limit reaching 5 fM of protein [166].

In an advanced GMR biochip designed to study DNA-DNA interactions rather than DNA probe-target detection, an on-chip GMR sensor offers real-time measurement of DNA binding and thermal denaturation on the solid-surface hybridization with temperature control [167]. Another study reported the detection of cell-free DNA (cf-DNA) fragments using an array of 30 spin-valve GMRs integrated on a portable biochip platform for cancer diagnostics [168]. Through surface modification of a GMR sensor with amino-terminated SiO_2_, the sensor was capable of detecting different bio-targets, such as amine-modified DNA and interlukin-6 (IL-6) antibodies using a 30-nm magnetic marker [169].

Immunosensors and protein-based sensors based on the principle of GMR have also been developed. A dose-response detection of the biomarker S100β with a detection limit as low as 27 pg/mL was prepared by increasing the active area using 300 nm magnetic nanoparticles and controlling the surface chemistry of the sensor [159]. Multiplex protein assays were reported to sense eight types of cancer-related cytokines in real time. The magnetic signal was amplified, and the detection concentration extended to the fM range by using 50 nm magnetic labels [158]. 

**Figure 5:**
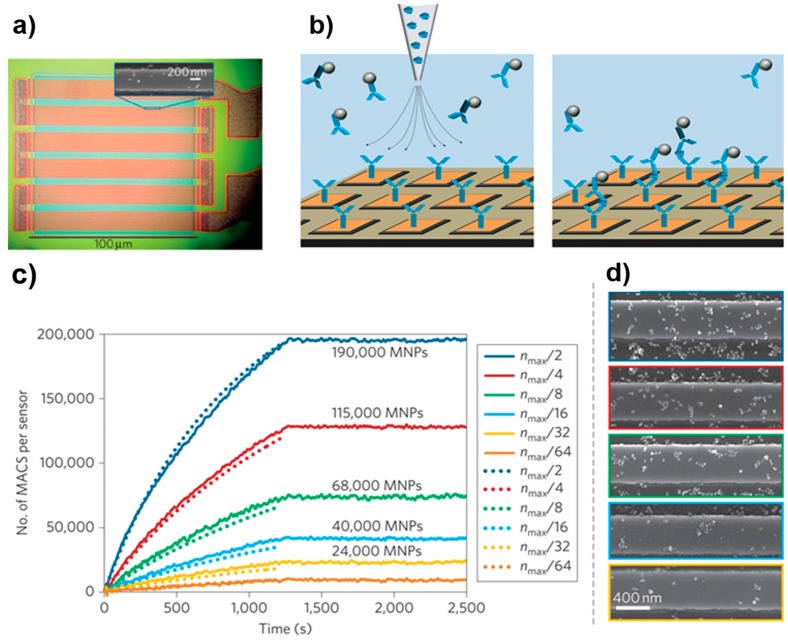
GMR applications: (**a**) optical image of GMR sensor architecture, where the upper right inset shows the SEM image of one strip with several bound MPs, (**b**) illustration showing the magnetic particle detection process, (**c**) real-time binding curve with fitting, and (**d**) SEM image of a section of the GMR sensor. Reproduced with permission from [12], copyright 2011 Nature Publishing Group.

As shown in Figure 5, a protein-based GMR sensor was further developed to quantify and monitor protein interactions and detect multiplexed proteins by functionalization with at least four different antibodies. The dynamic range can cover six orders of magnitude with a detection limit is as low as 50 aM [12]. A competition-based GMR assay was developed to detect unprocessed urine using smaller (12.8 nm) FeCo MPs [170]. Additionally, by combining a GMR sensor with magnetic tweezers, real-time DNA stretching and DNA-enzyme interactions were monitored [171]. The versatile applications of GMR sensors are summarized in Table 3.

TMR sensors are based on the tunnel magnetoresistance of a magnetic tunnel junction, which consists of a sandwich of two magnetic layers separated by an insulating layer (commonly MgO). TMR sensors offer a higher magnetoresistance ratio compared to GMR sensors and might provide higher sensitivity at low magnetic fields [15]. TMR sensors have been used for the detection of DNA hybridization and avidin binding in conjunction with 12 nm MnFe_2_O_4_ MPs [172]. Separately, an arrayed TMR sensor has been designed to detect target DNA by using 16 nm Fe_3_O_4_ and 50-nm polymer coated MPs [15]. Immunoassays with TMR sensors have been successfully developed to detect the liver cancer biomarker, α-fetoprotein (AFP) [173]. Meanwhile, the Hutter group has developed nanoscale TMR sensors to study the influence of the location of 1 μm MPs on the sensor surface for single-particle detection [174]. TMR sensors have been used to detect small quantities of iron oxide MPs in the complex lymphatic environment with good spatial resolution [175]. Additionally, a portable system has been integrated with a microfluidic apparatus as a lab-on-a-chip platform to detect pathogenic DNA below nM range sensitivity [176].

Planar Hall effect (PHE) sensors have been used to detect micro- and nano-sized MPs in biosensing [177]. The planar Hall effect is an exchange-biased permalloy planar sensor based on the anisotropic magnetoresistance effect of ferromagnetic materials. PHE sensors are capable of detecting micron-sized streptavidin-functionalized MPs [16]. A study of exchange-biased PHE sensors was investigated between −10 °C and 70 °C for temperature stability in biosensing experiments [178]. Miniaturized PHE sensors have been integrated with microfluidic systems and developed as room-temperature magnetorelaxometers to detect dynamic Brownian relaxation [179,180]. Recently, various PHE sensor architectures have been reported such as the spin-valve PHE with single-particle detection [181,182] and a PHE bridge sensor [183,184]. The latter gave higher signal compared to the cross geometry PHE sensor and has been used for on-chip DNA detection by measuring the Brownian relaxation of MPs [183].

### 4.2. Nuclear Magnetic Resonance (NMR)

Nuclear magnetic resonance (NMR) is a powerful magnetic phenomenon that can be used to identify, image, and detect molecules. In contrast to a surface-based sensor such as GMR, NMR and SQUID (described in the next section) are capable of measuring an entire volume of the sample [3,6,156]. NMR for detecting MPs is based on the change in nuclear spin relaxation induced by the magnetic field of MPs: the higher the transverse relaxation rate (R_2_), the higher the concentration of MPs.

One of the most promising applications of NMR in MP-based biosensing is magnetic resonance imaging (MRI) of localized MP-labeled molecules. The use of MPs in MRI is a non-invasive clinical method of molecular-level imaging. MPs play a dual role of both specific tags and contrast agents. The best known and commercially available MP-based contrast agents contain paramagnetic or superparamagnetic materials (e.g., manganese-based Mn-DPDP and magnetite-based Lumirem, respectively). Several reviews detail the design and functionality of MPs in MRI imaging with applications ranging from the molecular/cellular level to the organ level [7,8,37,38,39,74].

The multimodal labeling of MPs can overcome several limitations of single-purpose use found with optical bleaching and/or spatial resolution in optical imaging [161]. For example, multifunctional magnetofluorescent MPs in trimodal imaging combine MRI (iron oxide core), positron emission tomography (PET) sensing (chelator-ligand complexing the radiotracer Cu), and fluorescence (VivoTag-680) [206]. Moreover, MPs can play many functional roles, such as MRI contrast agents, radionuclide tracers, targeting moieties, and optical tags (e.g., fluorescence dyes, quantum dots, and NIR absorbers) [82,207].

**Figure 6:**
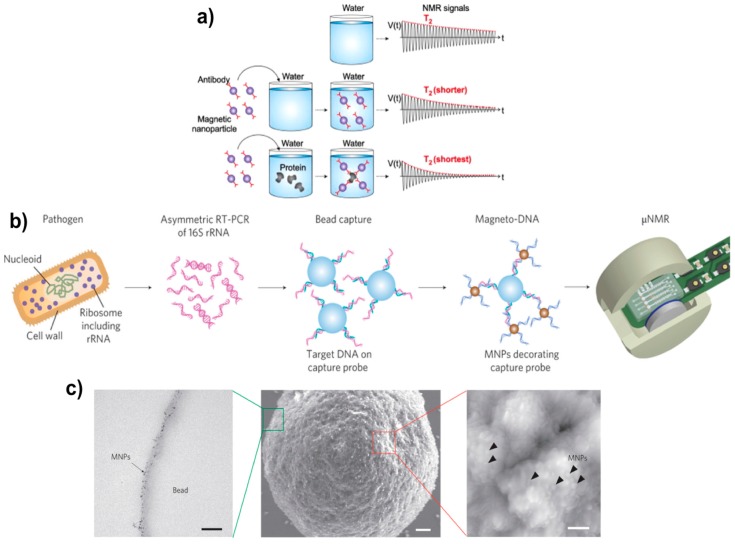
(**a**) Relaxation biosensor in NMR. (**b**) The magnetic switch application in magneto-DNA assay. (**c**) Capture bead and MP probe under TEM (left, scale bar, 100 nm), SEM (middle, scale bar 300 nm), and AFM (right, scale bar 100 nm). Reproduced with permission from [210], copyright 2014 American Chemical Society, and [40], copyright 2013 Nature Publishing Group.

The NMR principle has also been widely used in magnetic relaxation switches and MP-based relaxation sensors. The magnetic relaxation nanoswitches (Figure 6a) proposed by the Weissleder group are based on the self-assembly of 3–5 nm MPs to analyze molecules and cause a change in the spin-spin relaxation times of neighboring water molecules. The change in T_2_ relaxation is detected by a benchtop NMR relaxometer and can be employed to detect varieties of biomolecules such as DNAs, proteins, and enzymes [40,208].

With the improvement in electronics brought about by using integrated circuit (IC) chips and micrometer-sized detection coils, it is now possible to have portable and miniaturized NMR devices. The miniaturized NMR (μNMR) provides highly sensitive point-of-care diagnostics and can be integrated with microfluidic systems [209,210]. Application of μNMR in magnetic relaxation switches has advanced from molecular sensing to clinical diagnosis. Molecular sensing includes whole cells, proteins, DNA/mRNA, metabolites, drugs, viruses, and bacteria [211,212,213]. For example, Figure 6b,c illustrates a μNMR-based magneto-DNA assay that was used for the detection and phenotyping of bacteria [13]. In clinical studies, μNMR was successfully used to quantify and characterize lung tumor cells in primary blood samples and to monitor glioblastoma therapy from the blood samples of patients [208,212,214].

### 4.3. Superconducting Quantum Interference Device (SQUID)

SQUID has long been recognized as the most sensitive magnetometer, especially for low frequency applications [215,216]. The device is based on superconducting loops containing Josephson junctions. The current induced in the SQUID ring by an external magnetic flux gives rise to a change in voltage across the junction, which generates an output signal from the amplified voltage. SQUID-based biosensors are classified as relaxation-type, remanence-type, and susceptibility-type sensors [217]. In early studies, SQUID was used to detect ferromagnetic contamination in the lungs and other organs in the human body [218].

**Figure 7:**
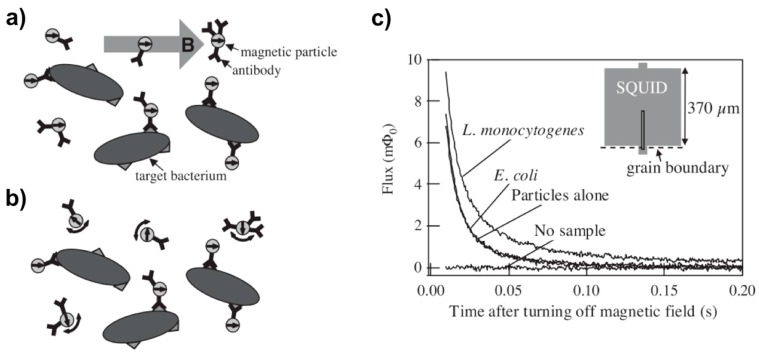
An example application of SQUID: (**a**) an applied magnetic field aligns the magnetic moment of MPs conjugated with an antibody, which are added into a bacteria-laden liquid suspension, (**b**) after removing the magnetic field, the unbound MPs have randomized magnetic moments due to Brownian rotation while the bound MPs slowly relax by Néel relaxation, and (**c**) magnetic decay signals including bacteria and unbound MPs. Insert: configuration of the YBCO SQUID. Reproduced with permission from [186], copyright 2004 National Academy of Sciences, U.S.A.

Most MP-based detection methods are based on measuring the change in magnetic field produced by the MPs. In the case of the relaxation sensor, bound MPs relax (Néel relaxation time of ~1 s for γ-Fe_2_O_3_ particles with 10 nm diameters) at a slower pace than unbound MPs (Brownian relaxation of ~50 μs for particles with 50 nm hydrodynamic diameter) [22]. Immunoassays based on Néel relaxation use direct current (DC) SQUID. For example, the Clarke group used a magnetic gradiometer-improved SQUID sensor to detect FLAG-tagged liposomes via immunoassay [22,219]. Figure 7 illustrates the use of SQUID to detect a magnetically-labeled *Listeria monocytogenes* bacteria suspension and to measure the binding rate between iron oxide MPs and the bacteria [186]. Another in vitro study explored the detection of iron oxide MPs conjugated to Her2-expressing MCF/Her2-18 cells (breast cancer cells) and the quantification of iron in tumors and the liver by SQUID-detected magnetic relaxometry [188]. A surface-based biosensor with high-T_c_ SQUID has also been used to measure the interaction of MP-labeled DNAs and antibodies with surface-immobilized receptors [187,192]. Immunoassays using SQUID can be studied by recording the magnetic response after switching off the magnetic field [220]. This type of magnetorelaxometry platform also allows kinetic studies of antibody-antigen reactions in whole blood samples [221]. Relevant liquid-phase immunoassays use the Brownian relaxation of labeled MPs to detect biotin and distinguish between bound and unbound MPs without requiring a washing step [193].

Remanence biosensors were also used to measure bound MPs in the absence of an external field after MPs were exposed to a magnetic field. The unbound MPs were removed as a ferrofluid [24]. Various reports in the literature measured MPs in ultra-dilute DNA solutions and multi-sample biological immunoassays with a detection limit as low as 30 pg of Fe_3_O_4_ MPs [24,189,222]. A SQUID-based magnetic immunoassay was used to distinguish between bound and unbound IgE on a surface without requiring any prior separation process [185]. In addition, the remanence measurement by SQUID can non-invasively track and identify MPs in human organs [217]. Using susceptibility measurements, the Yang group used immunomagnetic reduction methods to detect Alzheimer’s disease with a SQUID-based mixed-frequency AC magnetosusceptometer [190]. Other susceptibility measurements include the identification of in vivo ferrite MPs loaded on a tumor and 11 nm MPs in the lymph nodes of rats [21,191]. SQUID can also be used for MRI at ultra-low fields (mT). SQUID-based MRI in microtesla magnetic fields can image tumors with substantially greater T_1_-contrast as compared to high-field MRI. Through the use of MPs, the MRI can trace MP agglomerates (both bound and unbound MPs) and map magnetization decays by detecting their faster Néel relaxation in a magnetic field [223]. Liao and co-workers used high T_c_ SQUID to image field–dependent T_1_ relaxation of MPs in ferrofluids [224]. Furthermore, clinical magnetoencephalographic systems equipped with SQUID sensors can significantly enhance the measurement of neuronal currents when 25 nm Fe_2_O_3_ MPs are bound to a specific target [225].

### 4.4. Atomic Magnetometer (AM)

AM has been recently developed as a highly sensitive technique for measuring DC magnetic fields and rivals SQUID, with an advantage of no cryogenics required. The principle of AM is to measure the magneto-optical effects of spin-polarized atoms interacting with a probing laser beam in a magnetic field [226]. In AM measurements, a pump laser is typically used to polarize an alkali metal (K, Rb, or Cs) in the vapor phase. The subsequent precession of the polarized atoms in the magnetic field is detected by the optical rotation of a probe laser, which can be the same laser as the pump laser. Several different configurations of AM have been developed [157,226,227,228]. The sensitivity of an AM has been reported to be as low as 0.01 ft/Hz^1/2^ for a spin-exchange relaxation-free magnetometer [227]. Atomic magnetometers have been used to detect MPs in a variety of settings. For example, an AM was designed to detect a single 2-μm cobalt particle with continuous flow using microfabrication technology [228]. Recently, an important development centers on the scanning imaging method, which simultaneously provides spatial information and quantification of functionalized MPs; the resolution is approximately 20 μm with a 1-cm detection distance [229]. The imaging of MPs has been accomplished at physiological temperature; this measurement is suitable to study biological samples using an immunoassay [230]. Moreover, AM can be developed as a magnetic relaxometer for detecting medical samples such as targeted MPs and targeted cancer cells [23].

To reveal molecular information in MP-based biosensing, a technique described as force-induced remanent magnetization spectroscopy (FIRMS) has been recently invented [25]. FIRMS, with the integration of magnetic sensing using an atomic magnetometer, has been shown to be useful for studying specific molecular interactions between target–receptor biological pairs with unprecedented precision [25,229]. In this application, MPs are immobilized on a surface using a desired ligand-receptor pair to be studied, such as DNA duplexes or antibody-antigen pairs. When the applied mechanical force matches the binding force, bond dissociation gives rise to unbound MPs and their subsequent Brownian motion or removal; the reduced number of bound MPs remaining on the surface leads to a detectable reduction in the magnetic signal.

**Figure 8:**
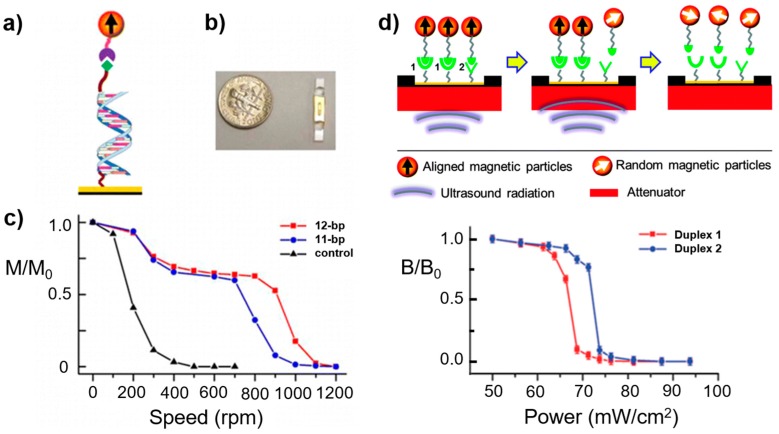
FIRMS studies using DNA: (**a**) a schematic of the FIRMS setup for DNA duplexes, (**b**) a photo of the sample well, (**c**) two DNA duplexes distinguished in a plot of magnetization versus force, and (**d**) the principle of acoustic radiation force (top figure) and an illustration binding profile of two DNA duplexes with the plot of magnetic field versus ultrasound power. Reproduced with permissions from [194], copyright 2014 Royal Society of Chemistry and from ref. [195], and copyright 2013 American Chemical Society.

The atomic magnetometer is the detector that measures the remanent magnetization from bound MPs on the surface. To distinguish between molecule-specific bindings, the applied external force on the receptor-tagged MPs is quantified. The external force is either a centrifugal force or an acoustic force as illustrated in Figure 8 [25,194]. Implementation of mechanical force in magnetic detection provides direct biological information on the binding strength of ligand-receptor pairs and can be used to investigate the precise interactions among biomolecules [25,30]. In FIRMS, MPs play three different functional roles: biological markers, magnetic signal carriers, and force transducers. 

The FIRMS technique has been used to study the binding force of the mouse IgG antigen-antibody interaction and DNA pairs as shown in Figure 8a–c [195,196]. In addition, the FIRMS technique provides information on the sequence and chiral selectivity of drug-DNA interactions [231]. A recent study using FIRMS was able to distinguish the human CD4 antibody-antigen binding force on antigen-functionalized surfaces and on CD4+ T-cell surfaces [197]. In another study, the FIRMS technique used the DNA binding force as a reference force to probe ribosome translocation with motor protein EF-G [198]. A FIRMS-derived technique, exchange-induced remanent magnetization (EXIRM), is based on the competitive binding between target microRNA and magnetically-labeled defect RNA and can be used for the label-free detection of microRNA. The exchanged microRNA can be detected and quantified through magnetic signal imaging even at a concentration as low as 10^4^ molecules [199].

### 4.5. Fluxgate Sensor

The fluxgate magnetometer has been commonly used to detect the change in magnetic fields for various disciplines such as archeology and oil-field surveys [29]. To detect MPs, the fluxgate sensor has been developed as a magnetorelaxometry technique [17]. Compared to a SQUID-type relaxometer, a fluxgate sensor offers room temperature detection and absolute magnetic fields [232]. The Schilling group has reported the potential of magnetic relaxation immunoassays by fluxgate magnetorelaxometry for various MP samples, including Fe_3_O_4_ MPs with or without a functionalized shell and MPs used in the degradation of hydrogels [232,233,234]. Binding assays based on fluxgate magnetorelaxometry have been used to study biotinylated agarose beads and bovine serum protein with streptavidin-functionalized MPs [202]. The complex susceptibility of CoFe_2_O_4_ MPs in gelatin solution has also been explored [235]. A micro-fluxgate-based biosensor was reported to detect carcinoembryonic antigen and α-fetoprotein in a double-antibody sandwich assay at a detection limit of 0.1 μg/mL of Dynabeads MPs and detectable antigen at 1 pg/mL [200]. Furthermore, fluxgate magnetometers have also been used to characterize the magnetization and relaxation dynamics of immobilized Fe_3_O_4_ MPs [236,237].

### 4.6. Frequency Mixing Magnetic Detection Techniques

Frequency mixing magnetic biosensors have been developed based on the detection of nonlinear magnetic materials. In this technique, magnetic fields of two different frequencies are used to excite the material, which gives a nonlinear response in which the frequency is a linear combination of the two separate excitation frequencies. Detection of the response indicates the presence of the magnetic material, for example, MPs [26,204]. This technique is specific to the applications of superparamagnetic MPs due to their nonlinear magnetic characteristics.

Applications of this technique have been demonstrated with the detection of many biological species, including c-reactive protein determination [238], *Francisella tularensis* in buffer and serum [239], *Yersinia pestis* in buffer and human blood serum [240], lipopolysaccharide from *Francisella tularensis* and F1 capsular antigen from *Yersinia pestis* [204], plant pathogens [241], and influenza A viruses on a nitrocellulose membrane platform [242]. Various types of immunoassays have been developed. For example, the Shin group reported a frequency mixing bioassay that employs two different MPs combined that work as magnetic separating and labeling in one effective analytical procedure; this study demonstrated the use of large biotinylated MPs for magnetic separation and small biotinylated MPs for magnetic quantification [205]. In another example, the planar frequency mixing magnetic technique employed 2D scanning imaging of superparamagnetic iron oxide MPs in a microfluidic platform for immunoassays. It successfully detected amyloid beta 42 (Aβ42), a promising biomarker of Alzheimer’s disease [243]. Furthermore, mixing-frequency magnetic detection has been reported to provide a volume-based magnetic sensing method. This method measured the Brownian relaxation of MPs as a way of monitoring the binding kinetics between protein G and its antibody [203].

### 4.7. Other Techniques

In addition to the techniques mentioned above, several alternative methods have been developed to detect magnetic particles. For example, susceptometry has been used to study the hydrodynamic size distribution of MPs using magneto-optical devices [244] and the reaction kinetics of MP clustering [245]. Magnetic binding assays were also reported to detect BSA adsorption using magnetic markers and a magnetic permeability meter [246]. Another related technique involves the air-core magnetometer, which is commonly used in biomedical applications [18]. In the inductive coil magnetic resonance detection, the effect of MP shape on signal amplification has been evaluated based on the magnetic susceptibility of oriented nanostructures [247]. Moreover, sensors based on domain wall movement have been developed to detect and localize MPs [19,248].

## 5. Summary and Perspectives

Magnetic particles (MPs) have been used in a wide range of diverse applications. In biomolecular sensing and imaging, MPs play a multifunctional role, including being a molecule-specific tag, a signal provider, or even a force dragger. Therefore, high magnetic strength, stable, and multifunctional MPs need to be judiciously designed for each technique targeted; the fabrication of such tailored MPs will lead to further enhanced biosensing capabilities. To expand the functionality of MPs and tailor them to each application, the composition and selection of suitable magnetic materials, surface-functional groups, and target molecules are all critical issues. This review covered the basic procedures for the custom synthesis of MPs for a range of biological applications and described several magnetic detection techniques (e.g., spintronic sensors, SQUIDs, AM-based sensors, and NMR sensors) that currently utilize MPs in biosensing applications. From the many studies reported, it is clear that size selection is one of the important factors in the choice of MPs in different types of magnetometer techniques. MPs smaller than 100 nm are widely used in applications based on the volumetric types of magnetometers, such as MRI imaging and SQUID-based immunoassays, where smaller particle sizes are needed (e.g., for cell uptake). On the other hand, large MPs (>100 nm) are commonly used in surface-based techniques for the quantification of properties, such as the GMR sensor and the FIRMS technique, where the larger particle give measurements with enhanced signal-to-noise ratios.

## Figures and Tables

**Table 1:** General Methods for the Synthesis of Magnetic Particles.

Method	Material	Size (nm)	Reference
Co-precipitation 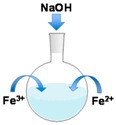	Fe_3_O_4_, Fe_2_O_3_	4–43	[42,43,44,45,46,47]
Thermal Decomposition 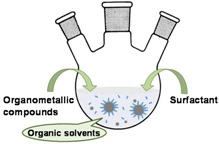	MFe_2_O_4_ (M = Fe, Co, Ni, Zn)	2–150	[48,49,50,51,52]
	Fe, Co, Ni		[53,54,55]
	FePt, FeCo, CoPt		[56,57,58,59,60,61]
Hydrothermal 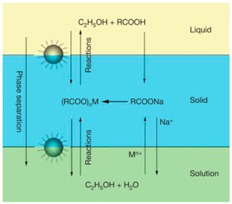	MFe_2_O_4_ (M = Fe, Co)	9–12	[62]
Polyol 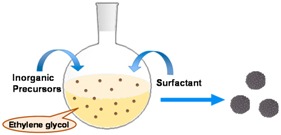	Fe_3_O_4_, FePt	3–10	[60,63]
	MFe_2_O_4_ (M = Fe, Co, Ni, Mn, Zn)	50–800	[64,65]
Emulsion/microemulsion/reverse micelles (self-assembly) 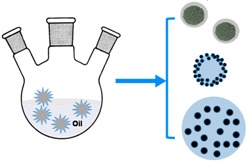	Fe_3_O_4_, MnFe_2_O_4_, Fe_3_O_4_/SiO_2_	2–200	[66,67,68,69]
	SiO_2_/Fe_3_O_4_ SiO_2_/Co SiO_2_/FePt	50–200	[66,70,71,72]
	Polymer/Fe_3_O_4_	1000–6000	[73]

**Table 2:** Common Bioconjugation Strategies.

Functional Ligands on MP	Reactive Molecules	Conjugated Product
**Carboxyl group**		
Carbodiimide coupling		
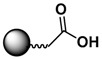	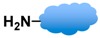	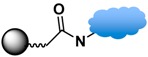
**Amino group**		
Homobifunctional cross-linking		a) Glutaraldehyde as a linker 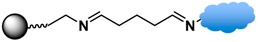
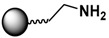	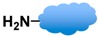	b) Disuccinimidyl suberate (DSS) as a linker 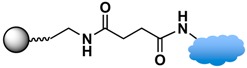
Maleimide coupling		
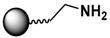	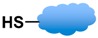	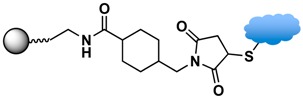
**Epoxide group**		
Direct reaction		
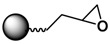	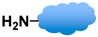	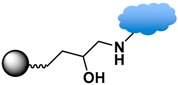
**Thiol group**		
Maleimide coupling		
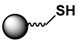	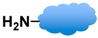	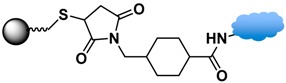
**Aldehyde group**		
Schiff-Base Condensation		
1) 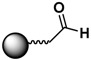	1) 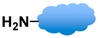	1) 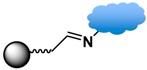
**Alkyne/azide group**		
Click reaction		
1) 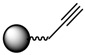	1) 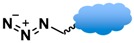	1) 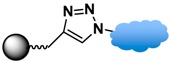
2) 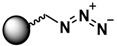	2) 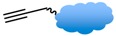	2) 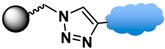

**Table 3:** MPs Detected by Various Magnetometer-Based Biosensing Techniques.

Method	Role of MPs	Type of MPs	Size	Biological Studies	Reference
GMR	Label, magneto-resistive effect on surface	Streptavidin-coated M270 and M280 (Dynal) *	2.8 μm	DNA	[84,162,163]
		Dynabeads Myone streptavidin	1 μm	Protein	[159]
		Bio-Adembeads Streptavidin	300 nm	Protein	
		Iron oxide (Bangs, CM021N)	350, 860 nm	DNA	[164]
		FeCo	100 nm	DNA	[165]
		Iron oxide MPs ^§^	30 nm	DNA, antibody	[169]
		Streptavidin-AF488-modified Cubic FeCo Nanoparticle	12.8 nm	Interleukin-6	[160,170]
		Streptavidin-coated superpara-magnetic beads (Micromod)	250 nm	DNA (cfDNA) fragments	[168]
		MACS †	50 nm	DNA, protein, biotin-streptavidin	[12,158,166]
TMR		MnFe_2_O_4_	12 nm	DNA	[172]
		MACS **	50 nm	DNA	[15]
		Fe_3_O_4_	16 nm		
		Fe_3_O_4_ (Ocean Nanotech) ***	20 nm	Protein	[173]
		Micromod Nanomag-D	250 nm	DNA	[176]
PHE		Amine-functionalized beads (Micromod)	50 nm	DNA	[183]
NMR	Label, relaxation	Dextran-coated iron oxide	21, 45 nm	DNA, protein	[185]
SQUID	Contrast agent, label, signal	Magnetite core (Quantum Magnetics, Madison, CT)	35 nm	Protein	[22]
		MACS ^†^	50 nm	Antibody-antigen, bacteria	[21,186,187]
		SHP-20 nanoparticles§	20 nm	Antibody	[188]
		Streptavidin-modified dextran-coated MPs (Meito Sangyo)	60 nm	DNA	[189]
		Polystyrene-coated particles (Dynal)	2.8 μm	DNA	
		Dextran-coated Fe_3_O_4_ (MagQu)	55–50 nm	Protein	[190]
		Fe_3_O_4_-coated with dextran (Meito Sangyo Co. Ltd.)	11 nm	Lymph node	[191,192]
		Self-made MPs	278, 322 nm	Biotin	[193]
		Ocean Nano-Technology	54 nm	Biotin	
		Bayer Schering Pharmacy, Resovist	58 nm	Biotin	
		R&D Systems, Mag-Cellect	112 nm	Biotin	
		Micromod, Nanomag D130	126 nm	Biotin	
		Micromod, Nanomag D250	150 nm	Biotin	
AM	Label	Ocean (SHP-28-50)	24 nm	Cell	[23]
	Label, signal, force transducer	Streptavidin-coated M280 (Dynal) *	2.8 μm	DNA, antibody-antigen	[25,194,195,196,197,198,199]
Flux-gate	Label and relaxation	Dynabead MyOne	1 μm	Antibody-antigen	[200,201]
		Streptavidin-coated MPs (Chemicell GmbH, Germany)	100 nm	Streptavidin-biotin BSA	[202]
Frequency mixing	Label and relaxation	Ocean SHP-35 nanoparticles	35 nm	Antibody-antigen	[203]
		Dynabead MyOne	1 μm	Antibody-antigen	[204]
		Dynabead M-280	2.8 μm		
	Signal	Nanomag^®^-D-spio-biotin	20 nm	Biotin-avidin	[205]
	Label, Separation	Micromod	500 nm		

* M270 and M280 (Dynal): 17–20% of iron oxide in a polymer-based microsphere. ^§^ Ocean nanoparticles (Ocean Nanotech; Springdale, AR, USA): single-core magnetite particles coated with a thin (~3–4 nm thick) layer of polymer and functionalized with carboxyl groups to enable conjugation to antibodies, with composite diameters ~20–30 nm. ^†^ MACS (Miltenyi Biotec, Germany): 55–59% Fe_2_O_3_ + 35–39% dextran + 2–10% biomolecular.
